# Efficacy of Diacetate Esters of Macular Carotenoids: Effect of Supplementation on Macular Pigment

**DOI:** 10.1155/2018/4632081

**Published:** 2018-03-01

**Authors:** Richard A. Bone, John T. Landrum, Anirbaan Mukherjee

**Affiliations:** ^1^Department of Physics, Florida International University, Miami, FL 33199, USA; ^2^Department of Chemistry and Biochemistry, Florida International University, Miami, FL 33199, USA

## Abstract

The accumulation of the carotenoids lutein, zeaxanthin, and mesozeaxanthin in the center of the human retina, and known as the *macula lutea* or macular pigment, is believed to protect the retina from age-related macular degeneration. Since the macular pigment is of dietary origin, supplements containing the relevant carotenoids are readily available. In this study, we compared the changes in macular pigment over a 24-week supplementation period for two groups of 24 subjects each assigned to either of two supplement formulations, 20 mg/day of lutein or 20 mg equivalent free carotenoids of a combination of diacetate esters of the macular carotenoids. The latter group responded with a larger increase (0.0666 ± 0.0481) in macular pigment optical density than the former group (0.0398 ± 0.0430), driven largely by the older subjects. The difference was statistically significant (*p*=0.0287). There was a general trend towards smaller increases in macular pigment for those subjects whose baseline value was high. However, the trend was only significant (*p* < 0.05) for subjects in the diacetate group. No differences in response could be attributed to the gender of the subjects. We also observed no indication that the use of statin drugs by a few of the older subjects influenced their responses.

## 1. Introduction

The macular carotenoids, or xanthophylls, lutein (L), zeaxanthin (Z), and mesozeaxanthin (MZ) are found throughout the human retina but are particularly concentrated in and around the fovea [1]. As both antioxidants and blue light blockers, they are believed to protect against degenerative retinal diseases such as age-related macular degeneration [2] and also, potentially, diabetic retinopathy [3] and retinitis pigmentosa/Usher syndrome [4]. Because the density of macular pigment in the retina responds positively to dietary supplementation with the macular carotenoids [5], it is appropriate to study different formulations with regard to their efficacy of absorption into the retinal tissues. The major source of L for the supplement industry is the marigold bloom (*Tagetes erecta*) where L is found in esterified form (56% lutein dipalmitate, 36% lutein dimyristate, and 8% lutein monomyristate [6]). However, the extraction and purification process, which typically involves alkaline saponification, results in free L. In order to obtain L esters from *T. erecta*, a food-grade solvent is employed [7]. Both free and esterified L are commercially available in the supplement form. A comparative study found that the bioavailability of the esterified form was higher than that for the free form as indicated by the increased uptake into the blood serum of free L [6]. (Following ingestion, the L esters are hydrolyzed prior to reaching the circulation.) The suggested reason for the higher bioavailability was the better dispersion, solubilization, and incorporation of L esters into micelles formed in the digestive process, compared with free L. However, this study contradicts a more recent crossover study by Chung et al. [8] in which no significant differences in serum response were observed when the subjects were taking free L or L ester supplements.

L esters can also be synthesized from free L, and the same is true for the other macular carotenoids, Z and MZ. The purpose of the present study was to determine the efficiency of absorption of a mixture of diacetate esters of L, Z, and MZ (Micro Mic™), relative to free L. Free L appears to be the most common commercially available supplement aimed at improving the health of the eye and was therefore considered an appropriate control for this study. Since the ultimate target tissue for the carotenoids is the retina, the efficiency of absorption was determined by directly measuring retinal levels of macular pigment.

## 2. Materials and Methods

### 2.1. Study Duration

Blood serum levels of carotenoids generally respond rapidly to carotenoid supplementation, reaching a plateau after about 4 weeks [9]. However, because the macular pigment responds more slowly, we adopted a 24-week supplementation period. Past studies have shown that during such a time period, significant changes in macular pigment optical density (MPOD) can generally be anticipated [9, 10].

### 2.2. Subject Demographics

A total of 48 subjects were recruited from the students, faculty, and staff at Florida International University. Subjects signed an IRB-approved informed consent form, and the study complied with IRB regulations as well as the Declaration of Helsinki. In order to compare possible age effects on the response to supplementation, we recruited subjects in two age ranges, 18 to 30, and over 50 years of age. The subjects were then split into two supplement groups: Group 1, consisting of 24 subjects, received 20 mg per day of Micro Mic in a L : MZ : Z ratio of 10 : 10 : 2; Group 2, also consisting of 24 subjects, received 20 mg per day of L (note that commercially available L contains approximately 5% of Z). With 24 subjects in each group and a desired power of 0.80, a significant difference between groups of 0.03 in the change of MPOD, with *σ* = 0.035, would be realizable. Subject demographics are summarized in Table 1. The study was a single-blinded study with the subjects not being informed which supplement they were receiving. Subjects were not asked to modify their diets, and an assessment of their normal dietary intake of xanthophylls was not included in this study.

### 2.3. Supplements

The supplements were provided in the form of identical looking gel caps that subjects were instructed to take with a meal, one per day, throughout the supplementation period. Each gel cap contained 20 mg of carotenoids (Table 1) in vegetable oil. In the case of the diacetate esters, 20 mg refers to the amount of free carotenoids, that is, not including the masses of the diacetate groups. For the lutein gel caps, 20 mg refers to the total amount of carotenoid, ∼95% of which was L and ∼5% was Z. Subjects were also given a 7-day pill organizer to aid in compliance and a schedule for future visits including dates for receiving refills. Compliance was determined by counting remaining gel caps at the end of the study and using this information to determine the number taken as a percentage of the number that should have been taken.

### 2.4. MPOD Measurements

MPOD was determined at baseline (week 0), at weeks 6, 12, and 18 and at the conclusion (week 24) of the supplementation period. The instrument employed was the mapcat SF™ [11], a heterochromatic flicker photometer that was used in a customized mode (cHFP). (“Customized” refers to using optimum flicker frequencies for the individual subject.)

MPOD was obtained in the right eye of each subject except for those whose vision was markedly better in the left eye. The subject viewed a small, circular stimulus, 1.5° in diameter and provided with crosshairs that alternated between blue and green lights provided by LEDs. The blue light is strongly absorbed by the macular pigment while the green light is only weakly absorbed. The result, generally, is a flickering appearance of the stimulus due to mismatched luminances. In order to determine the customized flicker frequency, the subject first viewed the stimulus with the green light switched off. Starting with a high blue light frequency (∼45 Hz), the subject gradually reduced the frequency until flicker was just perceived (critical fusion frequency, CFF). For the next stage of the test, the frequency was set to 2/3 of the CFF. With both the blue and green LEDs turned on, the subject altered the intensity of the blue light until, at equiluminance, flicker stopped or was minimized. Small adjustments to the frequency were made if the subject reported either a range of no flicker (frequency too high) or inability to eliminate flicker (frequency to low). The subject's blue light intensity setting at equiluminance reflected the amount of attenuation of the blue light, principally by the macular pigment and also, and increasingly with age, by the lens. The whole procedure was repeated with a larger, 15° stimulus with crosshairs for central fixation, and a default frequency set at 5/6 of the CFF. For this phase of the test, the subjects were asked to adjust the blue luminance to eliminate flicker around the periphery of the stimulus while ignoring the residual flicker at the center. Subjects made five repeat measurements for each part of the test, and the test was deemed acceptable if the standard error in the mean MPOD was less than 0.015. The MPOD and standard error were calculated automatically by a programmed microprocessor in the mapcat SF. The calculations are described in Bone and Mukherjee [11]. After a brief resting period, the entire test was repeated up to two more times. A weighted mean of the MPODs was calculated together with a standard error according to an algorithm published by Olive et al. [12].

### 2.5. Statistical Analysis

Results are expressed as means ± SD. Increases in MPOD resulting from supplementation were tested for significance using an independent-samples *t*-test (*α* = 2). Values of *p* < 0.05 were considered significant. Adjustments for potentially confounding factors such as body mass index were not included in the analyses.

## 3. Results

### 3.1. Retention and Compliance

All but two of the subjects completed the study satisfactorily. Of these, one of the younger subjects from Group 1 had to return to her homeland for personal reasons, and one of the older subjects from Group 2 developed severe difficulties with performing the cHFP test. Thus, reliable data were obtained from 23 subjects in Group 1 (11 young and 12 old), and 23 subjects in Group 2 (12 young and 11 old).

The average compliance, based on the percentage of pills taken, and associated standard deviation for each group are shown in Table 2.

### 3.2. Effect of Supplementation on MPOD

Positive changes in MPOD were obtained for the vast majority of the subjects. Eighteen subjects in Group 1 and fifteen subjects in Group 2 were considered as responders based on a change in MPOD greater than twice the standard error in the mean. Negative changes, usually very small, were observed for two subjects in Group 1, and four subjects in Group 2. In all but two of these cases, the presupplementation MPOD was high. The general effect of a high presupplementation MPOD on the change in MPOD will be discussed later.

**Figure 1:**
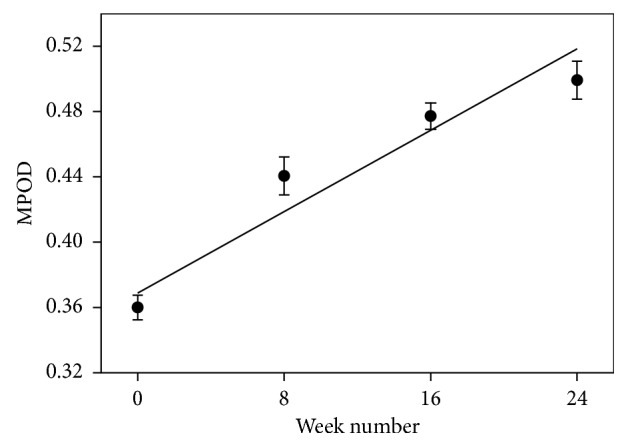
MPOD changes for an individual subject. This was a Group 1 subject taking 20 mg per day (equivalent free carotenoids) of diacetate esters of lutein, zeaxanthin, and mesozeaxanthin in a 10 : 10 : 2 ratio.

A robust response to supplementation is shown for one of the Group 1 subjects in Figure 1. It indicates, via the linear regression line, a remarkably linear relationship that was in fact typical of the majority of subjects. Note that the regression line is weighted using the reciprocal of the variance at each data point as the weighting factor. The average changes in MPOD ± SD from week zero to week 24 are shown for the two groups in Table 3. Also shown are the changes expressed as percentages of the week zero value and the rate of change in MPOD, that is, the slope of the regression line. Using two-tailed Student's *t*-test, we found a significant difference when comparing the change in MPOD for the two groups (*p*=0.0287).

### 3.3. Effect of Presupplementation MPOD on Change in MPOD

**Figure 2:**
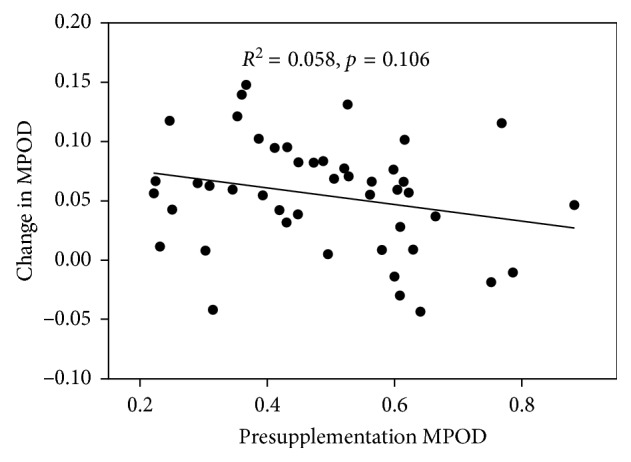
Change in MPOD as a function of presupplementation MPOD for all 46 subjects (23 taking 20 mg per day (equivalent free carotenoids) of diacetate esters of lutein, zeaxanthin, and mesozeaxanthin in a 10 : 10 : 2 ratio and 23 taking lutein).

There was a negative trend between the change in MPOD and the presupplementation MPOD for each group as well as for the combined groups.  Figure 2 shows the results for Group 1, the only one exhibiting a significant correlation. The slope of the regression line (−0.154) indicates by extrapolation that, on average, we might expect no change in MPOD with supplementation for a subject whose presupplementation MPOD was ∼0.93. For Group 2, and for Groups 1 and 2 combined, the slope of the regression line, the degree of correlation, and the significance were as follows: Group 2 (slope = −0.041, *R*^2^ = 0.033, *p*=0.41); Groups 1 and 2 combined (slope = −0.070, *R*^2^ = 0.058, *p*=0.106).

### 3.4. Effect of Age on Change in MPOD

Since the subjects were recruited in either of two age groups (18–30 = “young,” >50 = “old”), we were able to determine whether age was a significant factor in the MPOD response to supplementation. The MPOD response was quantified in three ways as indicated in Table 3: the overall change in MPOD, the percentage change in MPOD, and the rate of change in MPOD. The results ± SD are presented in Table 4 for the combined groups and for each individual group. Also included are the presupplementation MPODs and the *p* values for two-tailed *t*-tests to test for significant differences.

Since we found that the change in MPOD was significantly greater for Group 1 subjects when compared with Group 2 subjects, we made a similar comparison for the two age groups separately. The results are shown in Table 5 and indicate that the change in MPOD was greater for both old and young subjects in Group 1 compared with Group 2, but the difference in MPOD response was significant (∗) only for the older subjects (*p*=0.025).

### 3.5. Effect of Gender on Change in MPOD

Presupplementation MPOD was lower for the female subjects in the combined groups compared with the males but did not quite reach statistical significance. There were no differences between males and females for any measure of change in MPOD. The results are summarized in Table 6.

### 3.6. Effect of Statin Use on Change in MPOD

Statin use was restricted to the >50 year old subjects, six in Group 1 and three in Group 2. The data for the nonusers and users of this drug are summarized in Table 7. Included are the *p* values for Student's *t*-test for significant differences.

## 4. Discussion

Retention of subjects was high with only 2 subjects out of 48 being unable to complete the study. Likewise, Table 2 shows a very high, and almost identical, compliance for the two groups.

The results for Groups 1 and 2 revealed that the Micro Mic diacetate formulation assigned to Group 1 produced an average increase in MPOD that was 67% higher than that produced by the lutein formulation assigned to Group 2. The difference was statistically significant (*p* < 0.03). This result is consistent with that of Bowen et al. [6] who reported that levels of L in the serum were higher for subjects consuming the esterified form of L compared with those consuming free L. We also found that the change in MPOD bore a negative relationship with the presupplementation MPOD (Figure 2), albeit significant only for Group 1. However, the difference in the MPOD increases for Groups 1 and 2 could not be attributed to this finding since the average presupplementation MPODs were 0.499 and 0.477, respectively. We also considered the possible influence of statins since use of this drug may reduce L and Z levels in the serum [13] and, by extension, in the retina. However, because there were more statin users in Group 1 than in Group 2, we can rule out statin use as a contributor to the larger MPOD increase for Group 1.

There have been a number of other studies that examined the effect of lutein supplementation on MPOD. Direct comparisons are often difficult because of different doses, different supplementation periods, or different MPOD measurement parameters. For example, Nolan et al. [14] supplemented their subjects with 20 mg per day of a mixture of unesterified L, Z, and MZ (10 : 10 : 2). After 6 months, MPOD measured at 0.23° eccentricity increased by ∼0.06. Although this is very similar to the increase observed in the present study for subjects taking the esterified 10 : 10 : 2 mixture, MPOD measured with a 1.5° stimulus will always be significantly lower than that measured at 0.23° eccentricity [4]. In a study by Schalch et al. [15], one group of subjects received ∼20 mg/day of a mixture of L (∼10 mg) and Z (∼10 mg) for 6 months. MPOD measured by HFP with a 1° stimulus resulted in an average increase in MPOD of ∼15%. Again, this is similar to our own results which yielded ∼16% and ∼10% increases for Groups 1 and 2, respectively. Smaller increases in normal subjects were reported by Aleman et al. [4], whose subjects were supplemented with 20 mg/day of L for 6 months. The average increase was only 0.01 when measured by HFP with a 1° stimulus. However, the average increase was 0.07 for patients with retinitis pigmentosa or Usher syndrome.

As shown in Table 4, age was not a significant factor in determining the change in MPOD resulting from supplementation. This was true for Groups 1 and 2 individually or in combination. We also examined the data to see whether the larger change in MPOD for Group 1 subjects compared with Group 2 subjects was age-dependent. As seen in Table 5, both the young and old subjects in Group 1 had a larger change in MPOD than their counterparts in Group 2. However, the difference was largely due to the older subjects whose increase in MPOD was 144% higher for Group 1 than Group 2 subjects compared with 36% for the younger subjects. The difference for the older subjects was statistically significant (*p* < 0.05). Table 4 also contradicts a claim, popular among some in the supplement industry, that MPOD declines with age. Although not statistically significant, the average presupplementation MPOD for the combined groups was actually higher for the older subjects.

Prior to supplementation, the male subjects had, on average, a 17% higher MPOD than the female subjects, though the difference was not significant. Similar gender-based differences have been reported previously [16]. A possible reason could be that the diet of the male subjects resulted, on average, in a higher intake of xanthophylls than for the female subjects, but this was not assessed in this study. However, the increase in MPOD was almost identical for males and females.

With the use of statin drugs being commonplace among older persons, statin use was not included as an exclusion criterion. Nor did the study design include a comparison of MPOD responses between statin users and nonusers. Nevertheless, we noted that prior to supplementation, the statin users had, on average, a 22% higher MPOD than the nonusers. On the other hand, the change in MPOD was 29% lower for the statin users, but neither difference was statistically significant, probably owing to the small number (9) of statin users. Because these findings appear to contradict each other, we are unable to present evidence to either refute or support the earlier finding that statin use was associated with lower serum levels of L and Z [13]. Our results could also simply be a reflection of Figure 2, that is, subjects whose baseline MPOD is high generally have a smaller increase in MPOD.

The limitations of this study include the lack of a placebo group. However, our past experience has led us to conclude that MPOD changes over a 6 month period are insignificant when subjects are assigned to a placebo group [17, 18]. Also, we did not include an assessment of the subjects' dietary intake of xanthophylls which we would expect to influence their presupplementation MPOD. On the other hand, the supplements provided an approximately 10-fold increase in xanthophyll intake over the average dietary intake, so the subjects' diets would not be expected to influence changes in MPOD in any significant way. Lack of serum analysis of carotenoids in this report might also be considered a limitation; however, the target tissue for this study was the neural retina where the beneficial effects of the macular carotenoids are believed to occur. Finally, we did not use a mixture of unesterified L, Z, and MZ as our control. Therefore, it might be argued that the larger increase in MPOD observed for the diacetate group was a result of the inclusion of the zeaxanthin stereoisomers in the supplement rather than the esterification. We would argue against this possibility based on past observations, including our own, that neither Z nor MZ appears to produce as large a change in MPOD as L [15, 18, 19]. Nonetheless, we acknowledge the report by Loughman et al. who found that supplementation with all three macular carotenoids produced significant increases in MPOD whereas supplementation with L did not [20].

## 5. Conclusions

In conclusion, we have found that a combination of macular carotenoids in diacetate form was more effective at raising MPOD than unesterified L, especially in older subjects. This suggests that these particular esters may be more readily absorbed, that is, more bioavailable, than their unesterified counterparts.

## Figures and Tables

**Table 1:** Subject demographics.

Group	Number of subjects	Number in age groups		Male/female	Average age ± SD in age groups			Supplement
		18–30	>50		All	18–30	>50	
1	24	12	12	9/15	42.9 ± 22.1	22.0 ± 3.0	63.8 ± 8.1	Micro Mic (L : MZ : Z : = 10 : 10 : 2)
2	24	12	12	13/11	40.5 ± 18.9	22.9 ± 2.8	58.0 ± 8.3	Lutein (∼5% Z)

**Table 2:** Compliance.

	Percent compliance ± SD
Group 1	95.6 ± 6.1
Group 2	95.9 ± 5.4

**Table 3:** MPOD changes ± SD.

	Change in MPOD (AU)	% change in MPOD	Rate of change in MPOD (mAU/week)
Group 1	0.0666 ± 0.0481	16.2 ± 12.8	2.54 ± 2.03
Group 2	0.0398 ± 0.0430	10.4 ± 12.1	1.71 ± 1.83

AU = absorbance units; mAU = milliabsorbance units.

**Table 4:** Effect of age on MPOD.

	All groups, young	All groups, old	*p*
Presupplementation MPOD	0.440 ± 0.192	0.501 ± 0.142	0.18
Change MPOD	0.0617 ± 0.0479	0.0489 ± 0.0454	0.31
% change MPOD	18.0 ± 16.9	11.1 ± 11.4	0.075
Rate MPOD (mAU/wk)	2.46 ± 2.11	1.79 ± 1.79	0.20

	Group 1, young	Group 1, old	*p*

Presupplementation MPOD	0.510 ± 0.154	0.488 ± 0.110	0.70
Change MPOD	0.0669 ± 0.0509	0.0723 ± 0.0447	0.79
% change MPOD	16.0 ± 15.2	16.4 ± 10.7	0.94
Rate MPOD (mAU/wk)	2.53 ± 2.44	2.56 ± 1.68	0.97

	Group 2, young	Group 2, old	*p*

Presupplementation MPOD	0.466 ± 0.215	0.488 ± 0.169	0.78
Change MPOD	0.0491 ± 0.0454	0.0296 ± 0.0398	0.28
% change MPOD	13.6 ± 12.5	7.0 ± 11.2	0.20
Rate MPOD (mAU/wk)	2.09 ± 2.03	1.30 ± 1.58	0.31

**Table 5:** Change in MPOD for young and old subjects in Groups 1 and 2.

	Change in MPOD ± SD	
	Young	Old
Group 1	0.0669 ± 0.0509	0.0723 ± 0.0447
Group 2	0.0491 ± 0.0454	0.0296 ± 0.0398
*p*	0.39	0.025^∗^

**Table 6:** Gender effects on MPOD.

	Female	Male	*p*
Presupplementation MPOD	0.450 ± 0.154	0.528 ± 0.163	0.10
Change MPOD	0.0556 ± 0.0504	0.0538 ± 0.0440	0.90
% change MPOD	14.5 ± 14.5	12.0 ± 10.5	0.49
Rate MPOD (mAU/wk)	2.00 ± 2.19	2.26 ± 1.71	0.66

**Table 7:** Effect of statin use on MPOD.

	Nonstatin users	Statin users	*p*
Presupplementation MPOD	0.476 ± 0.163	0.536 ± 0.157	0.33
Change MPOD	0.0580 ± 0.0470	0.0413 ± 0.0467	0.35
% change MPOD	14.1 ± 12.9	10.0 ± 11.8	0.37
Rate MPOD (mAU/wk)	2.23 ± 2.02	1.72 ± 1.71	0.45
